# The role of prophylactic ibuprofen and N-acetylcysteine on the level of cytokines in periapical exudates and the post-treatment pain

**DOI:** 10.1186/2008-2231-20-30

**Published:** 2012-09-10

**Authors:** Maryam Ehsani, Ali-Akbar Moghadamnia, Samir Zahedpasha, Ghorban Maliji, Sina Haghanifar, Seyyed Mohsen Aghajanpour Mir, Narges Mousavi Kani

**Affiliations:** 1 Department of Endodontics, Dental Material Research Center, Faculty of Dentistry, Babol University of Medical Sciences, Babol, Iran; 2 Department of Pharmacology, Faculty of Medicine, Babol University of Medical Sciences, Babol, Iran; 3 Cellular and Molecular Biology Research Center, Babol University of Medical Sciences, Babol, Iran; 4 Department of Oral and Maxillofacial Radiology, Faculty of Dentistry, Babol University of Medical Sciences, Babol, Iran

**Keywords:** Periapical exudate, N-acetylcysteine, Ibuprofen, Cytokine, Pain

## Abstract

**Background:**

Periapical lesions are inflammatory diseases that result in periapical bone destruction because of host defensive–microbial disturbances.

**Objective:**

To evaluate the role of prophylactic ibuprofen and N-acetylcysteine (NAC) on the levels of tumor necrosis factor alpha (TNF- α), interleukin- 6(IL-6) and IL-17 and post-treatment pain level in chronic periapical lesions.

**Materials and methods:**

Eighty patients with chronic apical lesions less than 1 cm were randomly assigned to receive NAC tablets (400 mg), ibuprofen tablets (400 mg), NAC (400 mg)/ibuprofen (200 mg) combination and placebo 90 minutes prior to sampling. Periapical exudates were collected from root canals. TNF- α, IL-6 and IL-17 levels were determined by ELISA and post-treatment pain was assessed using a visual analog scale (VAS).

**Results:**

There was a significant difference in IL-6 level between ibuprofen group and placebo (p = 0.019). Significant difference in IL-17 level was observed between NAC/ibuprofen combination group and placebo (p = 0.043). Four hours after treatment, a significant difference was observed in VAS pain score between ibuprofen group and placebo (p = 0.017). Eight hours post-treatment, VAS pain score for NAC group was statistically lower than placebo group (p = 0.033). After 12 hours VAS pain score showed a significant decrease in NAC group compared to placebo (p = 0.049).

**Conclusion:**

The prophylactic ibuprofen and NAC failed to clearly reflect their effect on cytokines levels in exudates of chronic periapical lesions. On the other hand it seems that NAC can be a substitute for ibuprofen in the management of post endodontic pain.

## Introduction

Periapical lesions are inflammatory diseases that develop as result of root canal bacterial infections and may result in periapical bone destruction because of host defensive–microbial disturbances [1,2]. Neutrophil granulocytes, among infiltrating leukocytes, are the first line of defense which stimulate the migration of monocytes and lymphocytes [3]. Infiltrates of mononuclear cell, composed of antigen-presenting cells, T and B lymphocytes and their effectors are characteristic of chronic periapical processes [3]. Cytokines are low-weight messenger molecules between the host cells secreted by different immune cells and believed to have an important role in treatment and pathogenesis of many inflammatory diseases such as periradicular lesions [4,5]. Tumor necrosis factor alpha (TNF-α) has a wide range of pro-inflammatory and immunomodulatory effects on a number of different cell populations. It stimulates prostaglandin synthesis, bone resorption, and protease production by many cell types, including fibroblasts and osteoblasts. Extraproduction or inappropriate expression of TNF-α can lead to a variety of pathological conditions [6]. Interleukin- 6 (IL-6) has been traditionally considered to be a pro-inflammatory cytokine that may have a part in inflammatory process of periapical lesions [7]. IL-6 may also release locally in inflamed pulp and periradicular lesions, especially of chronic types [8]. IL-17 is the first member of an emerging family of inflammatory cytokines whose biological activities remain incompletely defined. IL-17 is produced exclusively by activated memory T-cells. IL-17, derived from T-cells, may play an important role in the initiation and maintenance of pro-inflammatory responses, and has recently been found to stimulate osteoclastic resorption [9,10]. For tooth pain, none steroidal anti-inflammatory drugs (NSAIDs) are the most frequently administered analgesics. Many studies have shown ibuprofen to be very effective in control or reducing dental pain [11,12]. Ibuprofen blocks the cyclo-oxygenase-1 (COX-1) and - 2 (COX-2) enzymes both together, with a highly effective analgesic and anti-inflammatory action for post endodontic pain [11]. However, many NSAIDs have undesirable side effects like gastrointestinal (GI) irritation, ulcers and bleeding. NSAIDs exacerbate some inflammatory responses, such as in inflammatory bowel disease [13-15]. Tugendreich et al. offered an interesting explanation for the mechanism by which certain NSAIDS could increase the production of inflammatory mediators [16]. They showed that the administration of several NSAIDs to rats resulted in the stimulation of gene expression similar to that observed when rats were exposed to LPS. They concluded that NSAIDs cause injury to the gastrointestinal system and lead to leakage of commensal bacteria and/or LPS into the circulation provoking a systemic inflammatory response. N acetylcysteine (NAC) is a derivative of the amino acid L-cysteine and is currently indicated for acute paracetamol overdose [17]. Pharmacological functions include repletion of intracellular glutathione stores, scavenging of toxic oxygen free radicals (both directly and indirectly via increased glutathione concentrations) and suppression of TNF production [18]. NAC is well known as a mucolytic agent and exerts anti-inflammatory activity [19,20]. The anti-inflammatory activity of NAC takes place through its ability to inhibit the expression and release of a variety of pro-inflammatory cytokines [21] and it down-regulates cytokine-stimulated expression of leukocyte adhesion molecules [22,23]. Simultaneous administration of NAC and diclofenac potentiates the NSAID drug anti-inflammatory effect and helps obtaining the same effect at lower drug levels [24]. The aim of this randomized clinical trial mainly was to evaluate the role of prophylactic ibuprofen and NAC on the levels of TNF- α, IL-6, IL-17 in chronic periapical lesions and evaluation of the post treatment pain.

## Methods

This double-blinded randomized clinical trial (Registration code of clinical trial: IRCT201008114547N1) was done on 80 subjects of different age and sex. Selection criteria included patients with good general health who had a recent panoramic radiograph. Exclusion criteria included: 1) systemic diseases such as diabetes, hepatitis, HIV infection, immunosuppressive chemotherapy, bleeding disorder, inflammatory or autoimmune diseases like Behçet’s syndrome, arthritis, and AIDS; 2) history and/or presence of other infections; 3) specific physiological condition such as pregnancy or menstruation; 4) periodontal diseases; 5) oral ulcers; 6) current smoking; 7) GI problems, such as peptic ulcer; 8) treatment with any medication in the preceding week. Following recruitment all subjects were given verbal and written information concerning the study and gave their written consent prior to the clinical examination. The study was approved by ethics review board of Babol University of Medical Sciences and was performed in agreement with the declaration of Helsinki. For careful evaluation, a periapical radiographic examination with bisecting-angle technique was performed after detection of periapical lesion in panoramic radiographic view of involved tooth. Two examiners (an oral and maxillofacial radiologist and an endodontist) evaluated the radiographs and determined the size of periapical radiolucent area by a ruler. Subjects with radiolucent asymptomatic periapical lesions with a diameter of less than 1 centimeter were included in the study. All of the subjects in the case group were asymptomatic at the time of sample collection without any history of previous exacerbation of periapical lesions. The teeth studied could be as follows: All single rooted teeth, distal root of mandibular molars and palatal roots of maxillary molars. Further, groups, each containing 20 subjects, were randomly assigned to receive either NAC tablets (400 mg), ibuprofen tablets (400 mg), NAC (400 mg)/ibuprofen (200 mg) combination and placebo (starch), all were packaged in identical 500 mg capsules with the same color and size and then encoded by a third person unaware of the study protocol. Each participant received a package containing 2 capsules and consumed it 90 minutes before sampling. The teeth were anesthetized with 2 % lidocaine with 1/100000 epinephrine and access cavity was prepared using an end-cutting fissure bur (Dentsply Maillefer, Ballaigues, Switzerland). The involved teeth were isolated with a rubber dam. Following the measurement of the working length, the root canal was enlarged to size 40 using K-Flexofile (Dentsply Maillefer, Ballaigues, Switzerland). After the root canal was dried with sterilized paper points, two size 40 paper points (Absorbent paper points, Kerr Manufacturing Co., Romulus, MI, USA) were subsequently inserted into the root canal close to the established working length and held for 30 s. The actively draining teeth were supposed to be excluded from the study. If the paper point withdrawn from the canal was dry, a thin endodontic file was used carefully to penetrate through the apical foramen to bring exudates into the root canal from the periapical area. The tooth was excluded from the study when blood was visible along the paper point. The wetted length of paper points was measured immediately. The volume of the fluid was calculated from a standard curve as described before [25] and expressed as μL. Both paper points were immersed into sterile Ependorf vials containing 300 μL phosphate buffered saline (PBS), vortexed for 1 min and stored at −70 ° C until time of assay. Routine treatment of the patient was then continued.

### Measurement of TNF- α, IL-6, and IL-17 levels

TNF- α, IL-6, and IL-17 were measured by ELISA. Assays were carried out in accordance with manufacturer’s instructions (eBioscience, Vienna, Austria). The amount of TNF- α, IL-6, and IL-17 were determined by reference to standard curves (0–1000 pg mL^–1^) constructed with each assay. The concentrations of IL-6, TNF- a, and IL-17 in each sample were calculated based on the dilutions and exudates volumes. The results were expressed as picograms per milliliter (pg mL^–1^) for cytokine concentration. The detection limit for TNF-alpha was 2.3 pg mL^–1^, 0.92 pg mL^–1^ for IL-6, and 0.5 pg mL^–1^ for IL-17 respectively.

### Pain recordings

Following the endodontic treatment, the patients were asked to record the level of pain and discomfort using a standard VAS scale and complete the postoperative questionnaires at pre-defined time points according to the following schedule: at 4, 8, 12, and 24 h after treatment [26]. Pain was defined as the presence of any degree of discomfort and was scored from 0 (no pain) to 10 (worst pain). Escape medication was included in the patients’ packets were (Darou Pakhsh Pharmaceutical Co., Tehran, Iran) for inadequate pain control from the trial medication.

### Statistical analysis

Cytokine levels in the periapical exudates were compared between groups by using Mann–Whitney *U* test. VAS pain scores were compared using one-way ANOVA post hoc Tukey test. A value of p < 0.05 was required for statistical significance.

## Results

### Assessment of TNF- α level

**Figure 1:**
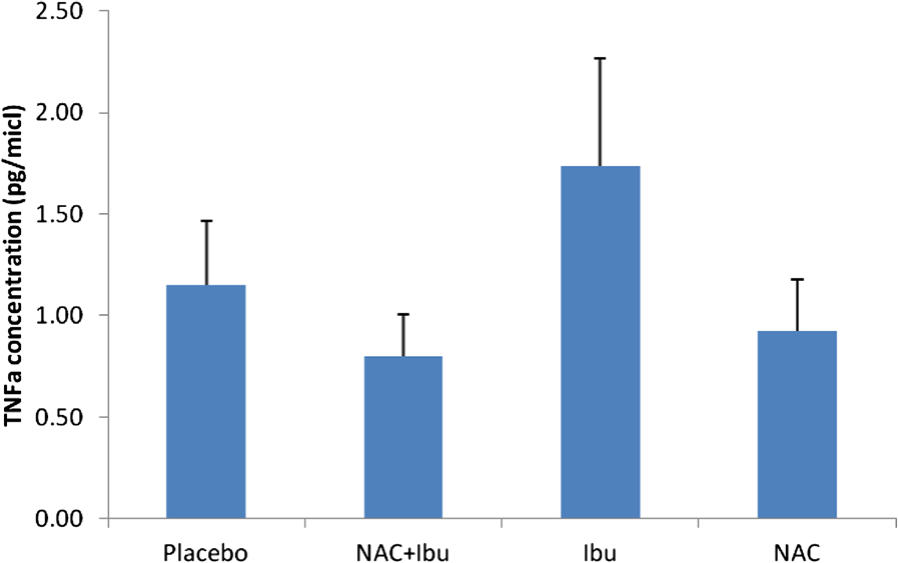
**Mean ± SEM of TNF- α concentration (pg ml**^**-1**^**) in the four study groups (n = 20).** No significant differences were seen between treatment groups compared to placebo. NAC; N-acetyl cysteine, Ibu; ibuprofen.

TNF- α was detected in all periapical exudates samples. However the difference between groups was not statistically significant regarding TNF- α levels (Figure 1).

### Assessment of IL-6 level

**Figure 2:**
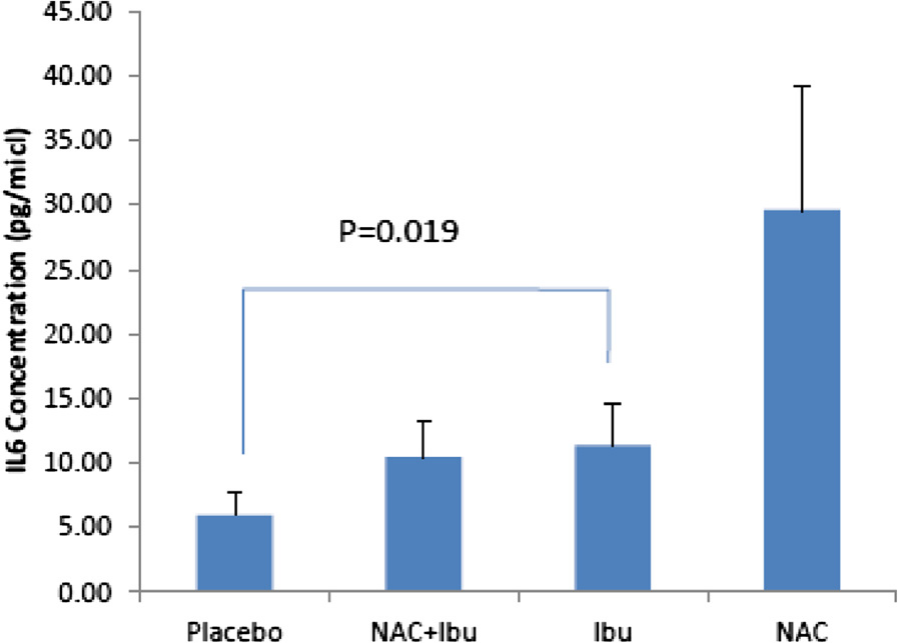
**Mean ± SEM of IL-6 concentration (pg ml**^**-1**^**) in the four study groups (with significant differerence between ibuprofen and placebo groups).**

IL-6 was not detected in all periapical exudates samples. There was a significant difference in IL-6 level between ibuprofen and placebo receiving groups (p = 0.019) (Figure 2).

### Assessment of IL-17 level

**Figure 3:**
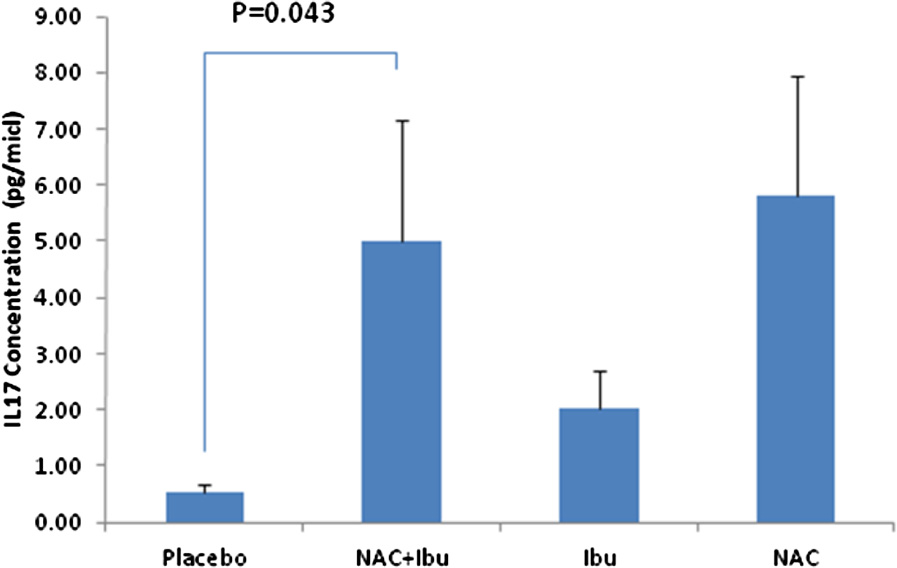
**Mean ± SEM of IL-17 concentration (pg ml**^**-1**^**) in the four study groups (with significant difference between NAC/Ibu and placebo groups).**

Undetectable levels of IL-17 were more pronounced compared to the other two cytokines. There was a significant difference in IL-17 level between NAC/ibuprofen combination group and placebo (p = 0.043) (Figure 3). No significant difference detected for ibuprofen and NAC treatment group compared to placebo.

### Assessment of pain level

**Figure 4:**
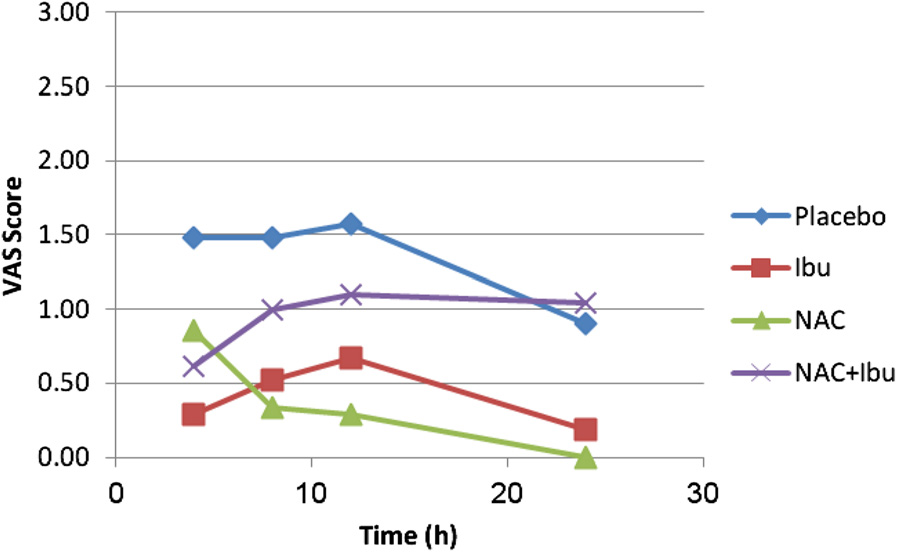
**Mean of VAS score changes by time (h) in four study groups (n = 20).** NAC; N-acetyl cysteine, Ibu; ibuprofen. The score for NAC group tends to zero after 24 hours post administration in comparison with other treatments.

No patients took any of the escape medication, which was provided in case of inadequate pain control. Figure 4 shows the mean VAS pain scores during 24 hours post treatment in four study groups. Considering VAS pain score, 4 hours after treatment, a significant difference was observed between ibuprofen and placebo receiving subjects (p = 0.017). Eight hours post-treatment, VAS pain score was statistically different between NAC group and placebo (p = 0.033). After 12 hours VAS pain score showed significant difference between NAC group and placebo (p = 0.049). No significant difference was found in VAS pain score at 24 hours after treatment between study groups (P > 0.05). Based on assessment of VAS pain scores by time after treatment, there were both inter-groups (p = 0.015) and intra-groups (p = 0.0001) differences in four study groups.

## Discussion

In this study we investigated the effect of prophylactic ibuprofen and NAC on the levels of TNF- α, IL-6 and IL-17 and post treatment pain level in chronic periapical lesions. Study drugs were given 90 minutes prior sampling. Ibuprofen is absorbed from the GI tract and its peak plasma concentrations are reached within about 1 to 2 hours after ingestion [27,28]. NAC is a thiol, a mucolytic agent and a source of sulfhydryl groups in cells and scavenger of free radicals as it interacts with ROS such as OH^·^ and H_2_O_2_[29]. Uses of NAC in different diseases including cancer, cardiovascular diseases, human immunodeficiency virus (HIV) infections, acetaminophen-induced liver toxicity and metal toxicity have been reviewed previously [30]. After an oral dose of NAC 200 to 400 mg the peak plasma concentration of 0.35 to 4 mg/L is achieved within 1 to 2 hours [31]. We collected the exudates from the root canals using noninvasive methods. There were no actively draining teeth in this experiment and none of the cases showed blood along the paper point. TNF- α was detected in all samples of periapical exudates but there were no significant differences between four study groups. Our results demonstrate the presence of IL-6 in the majority of tissue samples, but analysis of the data revealed that there was significant difference in IL-6 level only between ibuprofen group and placebo. This means ibuprofen 400 mg could not augment TNF-a to levels beyond the other groups but on the other hand increased IL-6 to much a level that was statistically different to placebo, but not to the other groups. Shahriari et al. [32] studied the effect of ibuprofen on IL-1β, TNF-α and PGE2 levels in periapical exudates. Ibuprofen was prescribed one tablet every 6 hour for three days and in the fourth day second samples were taken. Their results showed that PGE2 levels were decreased significantly in the case group following ibuprofen treatment comparing with the pre-treatment and placebo group. But there were no significant differences in IL-1β and TNF-α level between the two groups and in each group before and after treatment. In a study done by Spinas et al. [33] plasma levels of TNF- α, IL-1, and IL-6 were monitored after intravenous administration of Escherichia coli endotoxin with or without ibuprofen pretreatment to healthy volunteers. Pretreatment with ibuprofen caused a significant augmentation and temporal shift in cytokine elaboration. In a study by Sironi et al.[34]the effect of dexamethasone and two NSAIDs, ibuprofen and indomethacin, on the production of serum IL-6 and TNF levels in mice treated with endotoxin was investigated. Pretreatment with indomethacin or ibuprofen potentiated the production of both IL-6 and TNF. In the case of IL-6, indomethacin and ibuprofen were able per se to induce significant levels of this cytokine even in the absence of lipopolysaccharide (LPS). These data indicate that prostaglandins can physiologically provide a negative feedback regulation of IL-6 and TNF synthesis. Peristeris et al. [18] in their study investigated the effect of NAC on TNF production and LPS lethality in mice. The results indicated that oral administration of NAC inhibits the increase in serum TNF levels in LPS-treated mice and protects against LPS toxicity. The inhibition was not confined to the released form of TNF, since NAC also inhibited LPS-induced spleen-associated TNF. Hulten et al. [35] found that NAC attenuates TNF- α mRNA expression and secretion in macrophages from human lung transplant recipients and may be beneficial against transplant rejection. In a study carried out by Confalone et al.[36] NAC, a scavenger of reactive oxygen species, inhibited the activation of NF-κB and induction of IL-6 by TNF- α, being ineffective on IL- 1 β activity. It is apparent that the mentioned investigations are structurally different from our study and logically they cannot be compared. Our findings showed that undetectable levels of IL-17 were more pronounced compared to the other two cytokines. We found that NAC 400 mg/ibuprofen 200 mg combination increased the level of IL-17 to a level that a significant difference could be observed with control group. Data indicating the effect of these drugs on the level of cytokines in chronic periapical lesions are lacking in the literature. We measured post treatment pain level and discomfort using a standard VAS scale at pre-defined time points according to the following schedule: at 4, 8, 12, and 24 h after treatment. Four hours after treatment, a significant difference was observed in VAS pain score between ibuprofen group and placebo. Eight hours later, VAS pain score was statistically different between NAC group and placebo. After 12 hours VAS pain score showed significant difference between NAC group and placebo. No significant difference was found in VAS pain score at 24 hours after treatment between study groups. Hoffer et al. [24] found that LPS-induced prostaglandin E2 formation was significantly reduced by rofecoxib and by diclofenac, two NSAIDs. Adding NAC to each of these drugs enhanced the effect of the NSAIDs. They suggest the potentiation of the anti-inflammatory effect of COX inhibitors by their simultaneous administration with NAC, or obtaining the same anti-inflammatory effect at lower drug levels. In our investigation, no patients took any of the escape medication, which was provided in case of inadequate pain control; this could be due to the fact that in chronic periapical lesions pain is not a big issue following root canal therapy. From this study we can conclude that, the effect of ibuprofen and NAC on the periapical immunological events in chronic periapical lesions is still questionable, and more clinical trials are recommended to clarify these relationships. On the other hand, it seems that NAC can be a substitute for ibuprofen for post endodontic pain, but it is better to do more experiments especially in acute cases where an indication for analgesics seems more appropriate and rational.

## Competing interests

The author(s) declare that they have no competing interests.

## Authors’ contribution

ME participated in the design and conception of the study and has given final approval of the version to be published. AAM participated in the design of the study, performed the statistical analysis, and approved the English structure of manuscript to be published. SZ participated in the design of the study, patient management, drug delivery, acquisition of the data, drafted and revised the manuscript. GM participated in the design and conception of the study, helped in the immunological process of the samples and lab procedures and has given final approval of the version to be published.SH participated in the design of the study, and controlled the radiographic aspect of the study and has given final approval of the version to be published. SMAM participated in the acquisition of the data, performed the lab procedures and coordinated the immunological processes. NMK participated in the acquisition of the data, performed the lab procedures and coordinated the immunological processes. All authors read and approved the final manuscript.
